# Pädiatrische Anästhesieleistungen an deutschen Universitätskliniken: Eine deskriptive Analyse (2022–2024)

**DOI:** 10.1007/s00101-026-01666-2

**Published:** 2026-03-12

**Authors:** Armin Sablewski, Clemens Miller, Christiane E. Beck, Katharina Röher, Ehrenfried Schindler, Nina Zech

**Affiliations:** 1 Klinik für Anästhesiologie und Operative Intensivmedizin, Universitätsklinikum Schleswig-Holstein, Campus Kiel, Kiel, Deutschland; 2 Abteilung für Anästhesiologie, Orthopädische Kinderklinik Aschau im Chiemgau, Aschau im Chiemgau, Deutschland; 3 Klinik für Anästhesiologie und Intensivmedizin, Medizinische Hochschule Hannover, Hannover, Deutschland; 4 Klinik für Anästhesiologie, Universitätsklinikum Hamburg-Eppendorf, Hamburg, Deutschland; 5 Klinik für Anästhesiologie und operative Intensivmedizin, Universitätsklinikum Bonn, Bonn, Deutschland; 6 Klinik für Anästhesie und Kinderanästhesie, Krankenhaus Barmherzige Brüder – Klinik St. Hedwig, Regensburg, Deutschland

**Keywords:** Versorgungsforschung, Altersverteilung, Fallvolumen, Facharztweiterbildung, Zentralisierung der Versorgung, Health services research, Age distribution, Case volume, Specialist training, Centralization of care

## Abstract

**Hintergrund:**

Universitätskliniken nehmen eine Schlüsselrolle in der Forschung, klinischen Versorgung und Ausbildung ein. Die genaue Zahl der an Universitätskliniken durchgeführten Anästhesien bei Erwachsenen und Kindern ist derzeit nicht bekannt.

**Methode:**

Alle Ordinarien deutscher Universitätskliniken wurden per E‑Mail zur Teilnahme an einer Umfrage eingeladen. Für den Zeitraum von 2022 bis 2024 wurden jeweils die Anzahl an Anästhesieleistungen sowie Altersangaben (< 1, 1 bis 4, 5 bis 11, 12 bis 17, ≥ 18 Jahre) und der Status der ASA-Klassifikation der Kinder abgefragt. Primärer Endpunkt war der prozentuelle Anteil der pädiatrischen Anästhesieleistungen im Verhältnis zur Gesamtzahl. Sekundäre Variablen umfassten die altersabhängige Verteilung, den relativen Anteil, die ASA-Klassifikation sowie die fallvolumenabhängige Struktur pädiatrischer Anästhesien.

**Ergebnisse:**

Daten von 23 Universitätskliniken wurden ausgewertet (Rücklaufquote: 43,6 %). Durchschnittlich wurden pro Jahr und Klinik 24.910 ± 9325 Anästhesieleistungen erbracht. Der Anteil an Kindern betrug 13,6 % (davon 11,6 % < 1 Jahr und 31,8 % 1 bis 4 Jahre). Die Relation zwischen pädiatrischem Fallvolumen und Kinderanteil war positiv-linear (y = 0,0017x + 7,0785; R^2^ = 0,69). Die ASA-Klassifikation der Kinder verteilte sich auf 47,9 % ASA I, 28,5 % ASA II, 20,0 % ASA III, 3,3 % ASA IV und 0,2 % ASA V. Kliniken mit den größten Gesamtfallzahlen wiesen mit 16,5 % den höchsten Kinderanteil auf, einschließlich der meisten 0 bis 4 Jährigen (52,2 %) und der höchsten Anteile von Kindern mit ASA-IV- und ASA-V-Klassifikation (27,6 %).

**Diskussion:**

Zwischen den Universitätskliniken bestanden deutliche Unterschiede im Fallvolumen, jedoch zeigte sich eine Korrelation zwischen Gesamtleistungsumfang und Kinderanteil. Die Ergebnisse weisen darauf hin, dass pädiatrische Anästhesieleistungen zunehmend in großen Zentren konzentriert werden.

**Graphic abstract:**

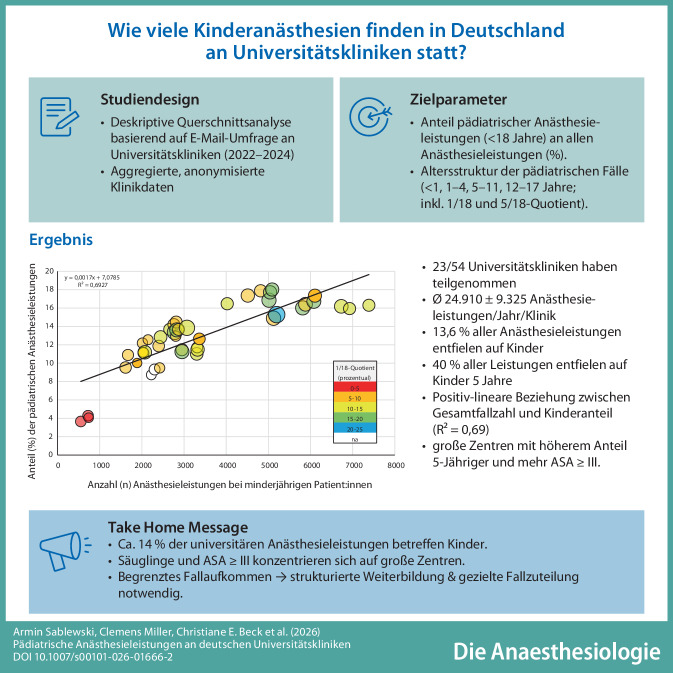

**Zusatzmaterial online:**

Die Online-Version dieses Beitrags (10.1007/s00101-026-01666-2) enthält das im Text aufgeführte Zusatzmaterial. Bitte scannen Sie den QR-Code.

## Abstract (en)

**Background:**

University hospitals play a key role in research, clinical care and medical education; however, the number of pediatric anesthetic procedures performed at German university hospitals, including complex cases requiring specialized expertise and those relevant for training remains unknown.

**Methods:**

All chairs of anesthesiology at German university hospitals were invited via email to participate in a survey. For the years 2022–2024, the number of anesthetic procedures, patient age groups (< 1, 1–4, 5–11, 12–17, ≥ 18 years) and the American Society of Anesthesiologists (ASA) classification of pediatric cases were collected. The primary endpoint was the percentage of pediatric anesthetic procedures relative to the total number of anesthetic procedures. Secondary variables included age distribution, relative proportion, ASA classification and case volume-dependent structure of pediatric anesthesia services.

**Results:**

In this study 23 university hospitals participated (response rate: 43.6%). On average, 24,910 ± 9325 anesthetic procedures were performed per hospital and year. The proportion of children was 13.6% (of whom 11.6% were < 1 year and 31.8% aged 1–4 years). The relationship between total case volume and the pediatric proportion was positive and linear (y = 0.0017x + 7.0785; R^2^ = 0.69). The ASA classification of children was distributed as follows: 47.9% ASA I, 28.5% ASA II, 20.0% ASA III, 3.3% ASA IV and 0.2% ASA V. Hospitals with the largest total caseloads had the highest pediatric proportion (16.5%), including the largest share of children aged 0–5 years (52.2%) and the highest proportions of ASA IV–V cases (27.6%).

**Discussion:**

There were marked differences in case volume among university hospitals; however, a clear correlation was observed between total anesthetic case volume and the pediatric proportion. The findings suggest that pediatric anesthesia services are increasingly concentrated in large centers.

Universitätskliniken haben den Auftrag der Lehre, Forschung und Krankenversorgung und erbringen in Deutschland täglich eine Vielzahl an anästhesiologischen Leistungen. Die konkrete Fallzahl an Anästhesieleistungen lässt sich lediglich schätzen, da eine systematische Erfassung bisher nicht erfolgt ist – auch nicht in der Kinderanästhesie. Diese Umfrage-Studie soll dazu beitragen, diese Lücke zu füllen, um eine fundierte Diskussion zu den Themen Ausbildung, Spezialisierung und Zentralisierung zu ermöglichen.

## Hintergrund und Fragestellung

Die anästhesiologische Versorgung umfasst ein breites Spektrum an zu behandelnden Patient:innengruppen, Eingriffsarten und Anästhesietechniken und ist integraler Bestandteil der perioperativen Medizin. In Deutschland ist nicht genau bekannt, wie viele Anästhesieleistungen jährlich erbracht werden. Die Deutsche Gesellschaft für Anästhesiologie und Intensivmedizin (DGAI) schätzt die Anzahl in deutschen Krankenhäusern auf rund 16 Mio. Anästhesieleistungen/Jahr [9]. Diese Schätzung basiert überwiegend auf Angaben zu operativen Prozeduren der fallpauschalenbezogenen Krankenhausstatistik, welche zuletzt für 2023 veröffentlicht wurden [19]. Operative Prozeduren sind jedoch nicht mit Anästhesieleistungen gleichzusetzen. Da auch kein nationales Register zur Erfassung aller Anästhesieleistungen hierzulande existiert, fehlen belastbare und öffentlich zugängliche Daten zur Fallzahlentwicklung von Anästhesieleistungen.

Dies betrifft auch spezifische Tätigkeitsfelder innerhalb des Fachgebiets, wie beispielsweise den Bereich der Kinderanästhesiologie. In der oben genannten Statistik lag der Anteil Minderjähriger bei etwa 3–4 %. Im internationalen Vergleich kursieren jedoch andere Zahlen: Das britische National Audit Project (NAP) berichtet von etwa 14 % aller stationären Anästhesien bei Kindern unter 18 Jahren [15]. Eine niederländische Studie berichtete einen Anteil von 18 % bei Kindern bis 16 Jahren [22]. Aufgrund der vergleichbaren Demografie dieser Staaten mit Deutschland scheint es naheliegend, dass die tatsächliche Anzahl der Anästhesieleistungen bei Kindern in Deutschland näher an diesen Prozentzahlen liegt.

Hierzulande werden Kinder vielerorts anästhesiologisch betreut, von ambulanten Praxen bis hin zu Kliniken mit unterschiedliche Größen und Strukturen. Auch in den Universitätskliniken werden Kinder anästhesiologisch betreut. Die universitären Abteilungen übernehmen dabei zentrale Ziele und Aufgaben wie die „Sicherstellung hochrangiger Forschung“, die „medizinische Spitzenversorgung“ sowie die „qualifizierte studentische Lehre und hochrangige strukturierte Weiterbildung graduierter Ärzte“ [2].

Die europäische APRICOT-Studie hat gezeigt, dass Kinder unter 3 Jahren ein erhöhtes perioperatives Risiko aufweisen, und dass die Erfahrung der Anästhesiolog:innen dieses Risiko deutlich verringert [13]. Universitätskliniken sind dabei sowohl Maximalversorger mit breitem Spektrum und hoher Anzahl an Gesamtanästhesieleistungen als auch Zentren für eine relevante Anzahl an Kinderanästhesien, einschließlich spezialisierter und komplexer Fälle, für die eine kinderanästhesiologische Expertise vorgehalten werden muss. Die zur Ausbildung verfügbaren Anästhesien bei Kindern bis zum vollendeten fünften Lebensjahr sind im Hinblick auf die für die Facharztweiterbildung geforderten Mindestzahlen von Interesse [5]. Aktuell sind weder der tatsächliche Bedarf an hochqualifizierten Anästhesiolog:innen mit spezieller kinderanästhesiologischer Expertise zur Versorgung komplexer Fälle noch die für die Ausbildung verfügbaren Fallzahlen zuverlässig quantifizierbar. Diese Aspekte verdeutlichen die Notwendigkeit präziser Versorgungsdaten, um sowohl die Qualität der Versorgung als auch die Weiterbildungsstrukturen in der Kinderanästhesiologie gezielt planen und weiterentwickeln zu können.

Ziel der vorliegenden Studie war es daher, ein realistisches Abbild der aktuellen Versorgungsstruktur an deutschen Universitätskliniken zu zeichnen und den Anteil pädiatrischer Patient:innen an allen erbrachten Anästhesieleistungen im Zeitraum 2022–2024 zu erfassen.

## Methoden

Die Datenerhebung dieser deskriptiven Querschnittanalyse erfolgte zwischen Mai und Juni 2025 per E‑Mail. Zur Teilnahme eingeladen wurden alle auf der Homepage der DGAI gelisteten Ordinarien für Anästhesiologie [8]. Aufgrund des Studiendesigns waren kein Ethikvotum und keine Studienregistrierung erforderlich.

### Datengrundlage und Datenverarbeitung

Die teilnehmenden Ordinarien übermittelten die Daten ihrer Universitätskliniken über durchgeführte Anästhesieleistungen für den Zeitraum 2022–2024 freiwillig, in aggregierter und anonymisierter Form per E‑Mail anhand einer optionalen Vorlage (Zusatzmaterial online, ESM_1 Blanko-Vorlage). Die übermittelten Daten wurden auf Plausibilität geprüft, wobei insbesondere Summenbildungen und Prozentwerte in den Tabellen kontrolliert, Gesamtzahlen mit Teilsummen abgeglichen sowie offensichtliche Unstimmigkeiten oder Ausreißer überprüft wurden. Bei Auffälligkeiten, unstimmigen oder unvollständigen Datensätzen wurden die jeweiligen Ordinarien gezielt erneut kontaktiert, um die Angaben zu verifizieren bzw. zu ergänzen.

Die erhobenen Daten umfassten jeweils für die einzelnen Jahre 2022, 2023 und 2024 die Gesamtzahl der erbrachten Anästhesieleistungen, die Anzahl an Anästhesieleistungen bei Minderjährigen (< 18 Jahre, aufgeschlüsselt in Altersgruppen) und den Status der ASA-Klassifikation der behandelten Kinder. Die Altersgruppen wurden anhand internationaler Standardisierung sowie der Vorgaben der Musterweiterbildungsordnung der Bundesärztekammer orientierend in die Altersgruppen < 1 Jahr, 1 bis 4 Jahre, 5 bis 11 Jahre und 12 bis 17 Jahre festgelegt [23].

Die tabellarischen Auflistungen der Universitätskliniken erfolgten in einer wertungslosen Nummerierung nach Eingang der Antwort. Die Namen der Universitätskliniken wurden anonymisiert.

### Studienendpunkte

Primärer Endpunkt war der prozentuelle Anteil der pädiatrischen Anästhesieleistungen im Verhältnis zur Gesamtzahl aller erbrachten Anästhesieleistungen in den Jahren 2022–2024. Sekundäre Variablen umfassten (I) die Aufteilung der pädiatrischen Anästhesien nach Altersgruppen, (II) den Anteil der pädiatrischen Anästhesieleistungen in Abhängigkeit von den Gesamtanästhesieleistungen, (III) die Verteilung der ASA-Klassifikationen bei Kindern und (IV) die Untersuchung von Anteil und Struktur pädiatrischer Anästhesien nach Fallvolumina der Universitätskliniken.

Zur Charakterisierung der Aufteilung nach Altersgruppen wurden die Quotienten „1/18“ und „5/18“ definiert. „1/18“ steht für den Anteil der unter Einjährigen an allen Kindern und „5/18“ für den Anteil der unter 5‑Jährigen an allen Kindern. Zur Untersuchung des Zusammenhangs zwischen Leistungsumfang einer Klinik und Anteil pädiatrischer Anästhesieleistungen wurden die Universitätskliniken anhand der Gesamtzahl an Anästhesieleistungen in Quartile eingeteilt (Q1 = geringster, Q4 = größter Leistungsumfang).

### Statistische Methoden

Eine Fallzahlkalkulation wurde nicht durchgeführt – die Studiengröße ergab sich aus dem Rücklauf an E‑Mails aller zur Teilnahme bereiten Ordinarien. Zur Darstellung des Anteils pädiatrischer Anästhesieleistungen im Verhältnis zum Umfang der gesamten Anästhesieleistungen wurden absolute und relative Häufigkeiten berechnet. Die Normalverteilung der Daten wurde mit dem Shapiro–Wilk-Test geprüft. Bei Normalverteilung werden Daten als Mittelwert ± Standardabweichung angegeben, anderenfalls als Median und Interquartilbereich. Der Zusammenhang zwischen der Gesamtzahl pädiatrischer Anästhesieleistungen und dem Anteil pädiatrischer Anästhesien am Gesamtanästhesieaufkommen wurde mittels linearer Regressionsanalyse untersucht.

Die statistische Auswertung, Datenverarbeitung und grafische Darstellung erfolgten mit Microsoft Excel (Office 2019, Microsoft, Redmond, WA, USA) und GraphPad Prism (GraphPad Software, San Diego, CA, USA).

## Ergebnisse

Die Umfrage wurde von 23 Ordinarien und ihren zugehörigen Kliniken (von insgesamt 54, Teilnahmequote 43,6 %) beantwortet.

### Anästhesieleistungen der Jahre 2022–2024

Im Zeitraum 2022–2024 erbrachten die 23 Universitätskliniken insgesamt 1.718.817 Anästhesieleistungen. Dies entspricht einer durchschnittlichen jährlichen Zahl von 24.910 ± 9325 Anästhesieleistungen/Klinik, mit einer Spannweite von 7650 bis 50.855 Leistungen. Im Jahresvergleich zeigte sich ein leichter Anstieg: Im Jahr 2022 wurden 24.151 ± 9150 Anästhesieleistungen dokumentiert, 2023 waren es 24.985 ± 9447 und 2024 schließlich 25.596 ± 9730.

### Pädiatrische Anästhesieleistungen der Jahre 2022–2024

In 16 Datensätzen war eine Differenzierung der Anästhesieleistungen zwischen Kindern (< 18 Jahre) und Erwachsenen (≥ 18 Jahre) möglich (Tab. 1). In den 7 anderen Fällen war diese Trennschärfe nicht gegeben, da die Altersaufteilung nicht nach der 18-Jahres-Grenze, sondern nach abweichenden Kategorien (z. B. nur bis 12 Jahre) erfolgte. Insgesamt entfielen im Zeitraum 2022–2024 13,6 % (165.924/1.219.633) der Anästhesieleistungen auf Kinder. Der Anteil variierte dabei zwischen 3,7 % und 18,0 %. 2022 lag der Kinderanteil bei 13,1 % (51221/391.756), 2023 bei 13,9 % (56.926/410.100) und 2024 bei 13,8 % (57.777/417.777).

**Tab. 1:** Verteilung der Anästhesieleistungen nach Altersgruppen an deutschen Universitätskliniken (2022–2024)

Universitätsklinik	Gesamt	≥ 18 Jahre	< 18 Jahre
Uni 1	63.672	54.999 (86,4 %)	8673 (13,6 %)
Uni 2	58.269	50.040 (85,9 %)	8229 (14,1 %)
Uni 3	62.800	Keine Angaben	Keine Angaben
Uni 4	23.459	Keine Angaben	Keine Angaben
Uni 5	50.010	47.999 (96,0 %)	2011 (4,0 %)
Uni 6	78.553	Keine Angaben	Keine Angaben
Uni 7	78.775	67.610 (85,8 %)	11.165 (14,2 %)
Uni 8	75.676	68.731 (90,8 %)	6945 (9,2 %)
Uni 9	86.458	71.314 (82,5 %)	15.144 (17,5 %)
Uni 10	85.067	75.496 (88,7 %)	9571 (11,3 %)
Uni 11	93.447	Keine Angaben	Keine Angaben
Uni 12	106.614	89.526 (84,0 %)	17.088 (16,0 %)
Uni 13	42.673	Keine Angaben	Keine Angaben
Uni 14	48.933	43.104 (88,1 %)	5829 (11,9 %)
Uni 15	77.361	64.008 (82,7 %)	13.353 (17,3 %)
Uni 16	60.975	53.269 (87,4 %)	7706 (12,6 %)
Uni 17	145.284	Keine Angaben	Keine Angaben
Uni 18	80.583	70.957 (88,1 %)	9626 (11,9 %)
Uni 19	105.392	88.286 (83,8 %)	17.106 (16,2 %)
Uni 20	52.968	Keine Angaben	Keine Angaben
Uni 21	53.836	48.308 (89,7 %)	5528 (10,3 %)
Uni 22	57.719	50.808 (88,0 %)	6911 (12,0 %)
Uni 23	130.293	109.254 (83,9 %)	21.039 (16,1 %)

### Altersverteilung der Kinder

Der größte Anteil der Anästhesieleistungen bei Minderjährigen in den Jahren 2022–2024 entfiel auf Kinder im Alter von 5 bis 11 Jahren (33,7 %), gefolgt von den Ein- bis 4‑Jährigen (31,8 %) und den 12- bis 17-Jährigen (24 %). Säuglinge unter einem Jahr machten 11,6 % aller Fälle aus (Tab. 2). Dabei variierte der Anteil der unter Einjährigen unter allen Kindern von 1,9–21,1 % bei einem Mittelwert von 10,4 ± 4,5 %. Die Anteile der unter 5‑Jährigen unter allen Kindern bewegten sich zwischen 31,7 % und 55,5 %, mit einem Mittelwert von 41,5 ± 5,6 %.

**Tab. 2:** Altersverteilung pädiatrischer Anästhesieleistungen (2022–2024)

Altersgruppe	Anteil (%)	*n*/*N*
< 1 Jahr	11,6	18.484/158.979
1–4 Jahre	31,8	46.986/147.814
5–11 Jahre	33,7	45.751/135.660
12–17 Jahre	24	32.564/135.660

Aufgrund inkonsistenter Datenlage war eine vollständige Kategorisierung nicht für alle Altersgruppen möglich. Eine detaillierte jahresbezogene Auswertung und die verfügbaren Datensätzen für die 1/18- und 5/18-Quotienten befinden sich ergänzend im Zusatzmaterial online.

### Anteil der pädiatrischen Anästhesieleistungen in Abhängigkeit von Fallzahlen und Gesamtanästhesieleistungen

**Abb. 1:**
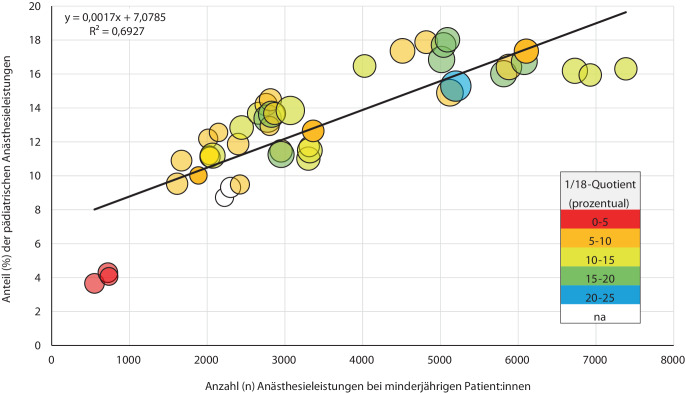
Altersstruktur und Anteil pädiatrischer Anästhesien in Abhängigkeit vom pädiatrischen Fallvolumen. Jeder Kreis repräsentiert einen Datensatz einer Universitätsklinik für jeweils ein einzelnes Jahr (2022, 2023 oder 2024). Die jahresweise Darstellung dient der Illustration der zeitlichen Konsistenz des Zusammenhangs; absolute Zahlen und Anteile werden im Text und in den Tabellen als über 3 Jahre aggregierte Werte berichtet. Die Farben kennzeichnen die 1/18-Quotienten entsprechend der in der Abbildung dargestellten Farbskala. Die Größe der Kreise entspricht dem 5/18-Quotienten

Mit zunehmender Gesamtzahl an pädiatrischen Anästhesieleistungen stieg der Anteil pädiatrischer Anästhesien zum Gesamtanästhesieaufkommen der Kliniken linear an (y = 0,0017x + 7,0785; R^2^ = 0,69; Abb. 1). An den Rändern der Regressionsgeraden zeigten sich insbesondere bei Kliniken mit sehr niedrigem oder sehr hohem pädiatrischen Anteil Abweichungen. Kliniken mit einem höheren Anteil sehr junger Kinder (1/18-Quotient ≥ 15 %) wiesen höhere pädiatrische Anteile auf (*grüne* und *blaue* Kreise in Abb. 1 weiter rechts auf der *x‑Achse*).

### ASA-Klassifikation

In die Auswertung des Status der ASA-Klassifikation der Kinder gingen die Datensätze von 13 Universitätskliniken ein. Von den verbleibenden 10 Universitätskliniken konnten 5 in diese Differenzierung nicht eingeschlossen werden, da die Daten auf Kinder bis 12 Jahre begrenzt waren, und 5 weitere aufgrund von unvollständigen Angaben zur ASA-Klassifikation.

112.266 Anästhesieleistungen bei Kindern (67,7 %) konnten einer ASA-Kategorie zugeordnet werden. Davon entfielen 53.726 Fälle auf ASA I (47,9 %), 32.020 auf ASA II (28,5 %), 22.506 auf ASA III (20,0 %), 3753 auf ASA IV (3,3 %), 261 auf ASA V (0,2 %) und 70 auf ASA VI (0,1 %).

### Anteil und Struktur pädiatrischer Anästhesien nach Fallvolumen

**Abb. 2:**
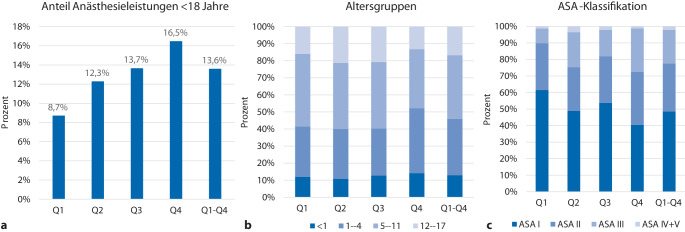
Anteil pädiatrischer Anästhesieleistungen sowie Alters- bzw. ASA-Verteilung nach Fallvolumen der Kliniken. Aufteilung der Universitätskliniken nach Gesamtzahl der Anästhesieleistungen in Quartile (Q1 = geringster, Q4 = größter Leistungsumfang); *Q1–Q4*: alle Kliniken zusammengefasst. **a** Anteil pädiatrischer Anästhesieleistungen an allen Anästhesieleistungen. **b** Altersverteilung der Kinder. **c** Verteilung der ASA-Klassifikation. ASA IV und ASA V wurden zur besseren Darstellung zusammengefasst. ASA VI wurde bei der Darstellung bei sehr geringer Fallzahl nicht berücksichtigt

Zur weiteren Charakterisierung der Aufteilung der ASA-Klassifikation bei Kindern wurden die 23 Universitätskliniken anhand ihrer Gesamtzahl an Anästhesieleistungen für die Jahre 2022–2024 in 4 Quartile eingeteilt. Die Quartilsgrenzen lagen bei 55.778 (25. Perzentil), 75.676 (Median) und 85.763 (75. Perzentil) Fällen. Die Zuordnungen der Kliniken zu den Quartilen (Q1–Q4) finden sich in den ergänzenden Tabellen im Zusatzmaterial online. Der Anteil pädiatrischer Anästhesieleistungen, der Alters- bzw. ASA-Verteilung nach Fallvolumen der Kliniken in Quartilen geclustert findet sich in Abb. 2. In Kliniken des vierten Quartils (größte Fallzahlen) zeigte sich mit 16,5 % der höchste Kinderanteil, begleitet von den höchsten Anteilen der 0‑ bis 4‑Jährigen mit 52,2 % und damit etwa 10 % mehr als in Q1 (41,5 %), Q2 (40,0 %) und Q3 (40,2 %) und den höchsten Anteilen von Kindern der ASA-IV- und ASA-V-Kategorien (Q1 10,2 %, Q2 24,7 %, Q3 18,1 % und Q4 27,6 %).

## Diskussion

Zwischen 2022 und 2024 entfielen an deutschen Universitätskliniken knapp 14 % der Anästhesieleistungen auf Kinder. Dabei war der Anteil an Kinderanästhesien umso höher, je mehr Anästhesieleistungen in einer Klinik erbracht wurden. Der Anteil der Kinder unter 5 und unter einem Jahr ist in Kliniken mit einem hohem Gesamtaufkommen an Anästhesieleistungen am höchsten.

Der Anteil pädiatrischer Anästhesieleistungen entspricht damit eher den international berichteten Daten aus Großbritannien und den Niederlanden [15, 22] als den auf operativen Prozeduren basierenden Fallzahlschätzungen der fallpauschalenbezogenen Krankenhausstatistik des Statistischen Bundesamtes [19]. Zwischen den verschiedenen Zentren zeigte sich eine gewisse Heterogenität, jedoch bestand eine positive Korrelation zwischen dem Anteil pädiatrischer Anästhesien in Abhängigkeit vom pädiatrischen Fallvolumen. Der 5/18-Quotient ließ eine relativ homogene Verteilung erkennen, wobei der Anteil aller Kinder unter 5 Jahre bei etwa 40 % lag. Im Gegensatz dazu wies der 1/18-Quotient eine deutlich größere Streuung auf, wobei der überwiegende Anteil der unter Einjährigen an Universitätskliniken mit einer hohen Anzahl an Gesamtanästhesieleistungen versorgt wurde (Abb. 1, *grüne* und *blaue Punkte* weiter rechts bei den größeren Fallzahlen zu verorten, und Abb. 2b). Dies legt nahe, dass insbesondere der Anteil von Säuglingen wesentlich vom Versorgungsprofil der jeweiligen Klinik abhängt, etwa durch die Konzentration spezialisierter Expertise auf Zentren mit Schwerpunkten wie der Behandlung angeborener Fehlbildungen [17]. Womöglich zeichnet sich in Deutschland ebenso eine Zentralisierungstendenz ab, wie sie in anderen Ländern wie Schweden [3] oder Großbritannien [21] bereits etabliert ist. Verschiedene Studien weisen darauf hin, dass eine spezialisierte Versorgung in Zentren besser für das Outcome der Kinder z. B. in Bezug auf Entlassungsraten, Reintubationen, neurologisches Outcome und sogar Mortalität ist [11, 12, 18]. Auch das Bundesministerium für Gesundheit plädiert in ihrer Krankenhausreform für das Bilden von spezialisierten Kliniken [6].

Eine Universitätsklinik in Deutschland führt durchschnittlich etwa 25.000 Anästhesieleistungen/Jahr durch. Rund 14 % davon sind Kinder, wobei davon wiederum etwa 40 % jünger als 5 Jahre sind (≈ 1300 Fälle jährlich bzw. 3 bis 4/Tag). Dieses Rechenbeispiel zeigt, dass nur wenige Anästhesiolog:innen täglich Kontakt mit diesen Kindern haben und es schwierig bzw. langwierig ist, eine Expertise in der Kinderanästhesiologie aufzubauen. Dass eine Vorhaltung von hoher Expertise generell von relevanter Bedeutung für die anästhesiologische Versorgung von Kindern ist, zeigte auch die APRICOT-Studie. Besonders die Zahlen der kardiopulmonalen Komplikationen in der Gruppe der Säuglinge waren sehr hoch [13]. Auch für die Gruppe von Früh- und Neugeborenen konnte eine hohe perioperative Morbidität und Mortalität gezeigt werden, die auf direkt anästhesiologische Faktoren wie Hypotonie, Anämie und Hypoxämie zurückzuführen waren [10]. Die aktuellen Richtlinien des American College of Surgeons (2021) empfehlen sogar, dass bei allen Kindern unter 24 Monaten primär ein:e Anästhesiolog:in mit spezieller kinderanästhesiologischer Expertise eingesetzt werden muss, sowie bei Kindern ≤ 5 Jahre oder mit ASA-Status ≥ III eingesetzt werden sollte [1]. Diese Empfehlungen unterstützen Initiativen wie das „Fellowship Kinderanästhesie“, welche eine strukturierte Weiterentwicklung von Fachärzt:innen in die Richtung spezieller Kinderanästhesiologie fördern möchten [16].

Besonders wertvoll sind diese Rechenbeispiele im Hinblick auf die Frage einer ausreichenden Anzahl von Anästhesieleistungen bei Kindern unter 5 Jahren zur Facharztweiterbildung gemäß den Anforderungen der Musterweiterbildungsordnung. Das tatsächliche Fallaufkommen wird durch Dienstpläne, Urlaubszeiten und operative Auslastung zusätzlich eingeschränkt. Hinzu kommt, dass die pädiatrischen Fälle nicht gleichmäßig über die Kliniken verteilt sind und komplexere Eingriffe – insbesondere bei Säuglingen oder Kindern mit ASA-Status  ≥ III – für unerfahrene Weiterzubildende nur bedingt geeignet sind. Durch die sinkenden Geburtenraten ist künftig mit einer weiteren Abnahme pädiatrischer Patient:innen zu rechnen [20]. Dies unterstreicht, dass die Ausbildungsreserve im universitären Umfeld knapp ist und derzeit v. a. zur Qualifizierung der eigenen Ärzt:innen in Weiterbildung genutzt werden kann. Ein in der Praxis gelebter und gängiger Weg ist es, durch Doppelbesetzung (erfahrene:r Anästhesist:in und Anästhesist:in in Ausbildung) Sicherheit und Ausbildungsqualität zu gewährleisten. Daraus ergibt sich die Notwendigkeit einer gezielten Fallzuteilung sowie strukturierter Rotationen, um eine ausreichende Exposition unter Supervision sicherzustellen.

Neben den Universitätskliniken existieren in Deutschland zahlreiche nichtuniversitäre Krankenhäuser und ambulante Praxen mit ausgewiesener Expertise in der Kinderanästhesiologie, in denen beispielsweise auch Kinder mit komplexen Fehlbildungen behandelt werden. Die Übertragung der Ergebnisse dieser Umfrage auf Fragen der Ausbildung und Expertise in der Gesamt-Kinderanästhesiologie ist daher nur eingeschränkt möglich. Dazu müsste der Anteil der universitären Einrichtungen an der Gesamtweiterbildung erfasst werden. Künftig wäre es daher sinnvoll, die Umfrage auf weitere Kliniken und den ambulanten Bereich mit einer kinderanästhesiologischen Versorgung auszuweiten, um zu prüfen, welche Versorgungsleistungen, Krankheitsschwere und Ausbildungskapazitäten dort bestehen.

Im universitären Umfeld wurden knapp 80 % der Kinder mit einem niedrigen perioperativen Risiko (ASA I 47,9 %, ASA II 28,5 %) eingestuft. In der APRICOT-Studie mit einer gemischten Kohorte aus universitären und nichtuniversitären Einrichtungen hatten etwa 60 % der eingeschlossenen Kinder den Status ASA I und 28 % ASA II [13]. Für Universitätskliniken ist beschrieben, dass häufig komplexe Fälle und Patient:innen mit chronischen Erkrankungen und damit höhere ASA-Stufen betreut werden [4]. Die hier vorliegenden Daten zur ASA-Klassifikation spiegeln das nicht wider. Möglicherweise beteiligen sich Universitätskliniken stärker an der pädiatrischen Regelversorgung als es bei Erwachsenen der Fall ist. Auch möglich erscheint ein relevanter Anteil gesunder Kinder, die wegen der Komplexität der Eingriffe universitär versorgt werden müssen. Eine falsche Anwendung der ASA-Klassifikation für Kinder scheint eine weitere Möglichkeit der Unterpräsenz hoher ASA-Klassifikationen zu sein [7, 14].

### Limitationen

Die Datenerhebung erfolgte retrospektiv und basierte auf freiwillig übermittelten Routinedaten, sodass eine externe Validierung nicht möglich war. Kleinere, nicht systematisch dokumentierte Anästhesieleistungen (wie z. B. anästhesiologisch begleitete innerklinische Transporte, Anlage von Gefäßzugängen, Hilfe bei Punktionen etc.) könnten u. U. nicht erfasst sein, sodass die tatsächlichen Zahlen womöglich etwas höher liegen.

Außerdem ist unklar, welche Art von Eingriff jeweils als „Anästhesieleistung“ erfasst wurde. So können in einer Klinik 100 Kinderherzoperationen und in einer anderen 100 Leistenhernien gleichermaßen als 100 Anästhesieleistungen gezählt werden. Da die Leistungen nicht nach Komplexität unterschieden werden, spiegelt die reine Anzahl nicht das Risikoprofil und den Ressourcenbedarf wider. Vor diesem Hintergrund haben die Autor:innen bewusst den Begriff „Anästhesieleistungen“ und nicht „Anästhesien“ oder „Narkosen“ gewählt.

## Fazit für die Praxis

- Rund 14 % aller Anästhesieleistungen an deutschen Universitätskliniken entfallen auf Kinder. Von diesen sind etwa 40 % unter 5 Jahre alt.
- Zentren mit einer höheren Gesamtanästhesieleistung versorgen mehr als 50 % Kinder unter 5 Jahren. Ihr Anteil an Säuglingsnarkosen ist besonders hoch und kann ein Hinweis auf Zentrumsbildung sein.
- Kliniken mit höheren Fallzahlen behandelten tendenziell mehr Kinder der ASA-Klassifikation 3 oder höher.
- Universitätskliniken müssen für Säuglinge und Kinder mit einer hohen ASA-Klassifikation eine ständige spezielle kinderanästhesiologische Expertise vorhalten.
- Das Gesamtaufkommen an für die Facharztweiterbildung geeigneten Kindernarkosen ist mit 3 bis 4/Tag über alle Zentren hinweg gering.
- Das begrenzte Fallaufkommen pädiatrischer Anästhesien erfordert deswegen eine gezielte Fallzuteilung und strukturierte Rotationskonzepte, um eine ausreichende Weiterbildung sicherzustellen.

## Supplementary Information

ESM 1_Blanko-Vorlage

ESM 2_Zuordnungen der Kliniken zu den Quartilen

ESM 3_Altersverteilung der Anästhesieleistungen

## Data Availability

Alle während dieser Studie generierten oder analysierten Daten sind in diesem veröffentlichten Artikel und den dazugehörigen ergänzenden Materialien enthalten.
