# Supplementary material for: Pediatric anesthesia services at German university hospitals: A descriptive analysis (2022–2024)
Source: Anaesthesiologie. 2026 Mar 12;75(6):413–21. [Article in German] doi: 10.1007/s00101-026-01666-2 (PMC13226350; doi:10.1007/s00101-026-01666-2)
Supplement: Supplementary file 3 — ESM 3_Altersverteilung der Anästhesieleistungen [file 101_2026_1666_MOESM3_ESM.docx]

# Zusatzmaterial zum Beitrag „Pädiatrische Anästhesieleistungen an deutschen Universitätskliniken – eine deskriptive Analyse (2022–2024)“ von Armin Sablewski, Clemens Miller, Christiane E. Beck et al. (2026) in *Die Anaesthesiologie.*

Beitrag und Zusatzmaterial stehen Ihnen auf www.springermedizin.de zur Verfügung. Bitte geben Sie dort den Beitragstitel in die Suche ein.

Altersverteilung der Anästhesieleistungen bei Kindern an deutschen Universitätskliniken (2022–2024).

| **Universität** | **<18 (n)** | **<1 (n/%)** | **1-4 (n/%)** | **5-11 (n/%)** | **12-17 (n/%)** |
| --- | --- | --- | --- | --- | --- |
| Uni 1 | 8673 | 1366 (15.8%) | 2854 (32.9%) | 2762 (31.8%) | 1691 (19.5%) |
| Uni 2 | 8229 | 756 (9.2%) | 2555 (31.0%) | 3126 (38.0%) | 1792 (21.8%) |
| Uni 3 | Keine Angabe | 1406 | 2887 | 3792 | Keine Angabe |
| Uni 4 | Keine Angabe | 750 | Keine Angabe | Keine Angabe | Keine Angabe |
| Uni 5 | 2011 | 53 (2.6%) | 643 (32.0%) | Keine Angabe | Keine Angabe |
| Uni 6 | Keine Angabe | Keine Angabe | Keine Angabe | 3641 | Keine Angabe |
| Uni 7 | 11.165 | 1320 (11.8%) | Keine Angabe | Keine Angabe | Keine Angabe |
| Uni 8 | 6945 | 2433 (35.0%) | | 2514 (36.2%) | 1998 (28.8%) |
| Uni 9 | 15.144 | 2571 (17.0%) | 4375 (28.9%) | 4407 (29.1%) | 3791 (25.0%) |
| Uni 10 | 9571 | 1351 (14.1%) | 2958 (30.9%) | 3046 (31.8%) | 2216 (23.2%) |
| Uni 11 | Keine Angabe | 934 | 4526 | 5979 | Keine Angabe |
| Uni 12 | 17.088 | 3006 (17.6%) | 5536 (32.4%) | Keine Angabe | Keine Angabe |
| Uni 13 | Keine Angabe | 920 | 2208 | Keine Angabe | Keine Angabe |
| Uni 14 | 5829 | 385 (6.6%) | 1675 (28.7%) | 2136 (36.6%) | 1633 (28.0%) |
| Uni 15 | 13.353 | 1285 (9.6%) | 4411 (33.0%) | 4318 (32.3%) | 3339 (25.0%) |
| Uni 16 | 7706 | 554 (7.2%) | 2326 (30.2%) | 2625 (34.1%) | 2201 (28.6%) |
| Uni 17 | Keine Angabe | 2423 | 7443 | 8295 | Keine Angabe |
| Uni 18 | 9626 | 809 (8.4%) | 2953 (30.7%) | 3628 (37.7%) | 2236 (23.2%) |
| Uni 19 | 17.106 | 1349 (7.9%) | 6576 (38.4%) | 6087 (35.6%) | 3094 (18.1%) |
| Uni 20 | Keine Angabe | Keine Angabe | Keine Angabe | 4696 | Keine Angabe |
| Uni 21 | 5528 | 351 (6.3%) | 1569 (28.4%) | 1937 (35.0%) | 1671 (30.2%) |
| Uni 22 | 6911 | 688 (10.0%) | 2249 (32.5%) | 2271 (32.9%) | 1703 (24.6%) |
| Uni 23 | 21.039 | 2640 (12.5%) | 6306 (30.0%) | 6894 (32.8%) | 5199 (24.7%) |

Für die Auswertung des 1/18-Quotienten standen Datensätze aus 15 Kliniken über drei Jahre hinweg zur Verfügung, was insgesamt 45 Datensätzen entspricht. Für den 5/18-Quotienten lagen Daten von 15 Kliniken vor, sodass insgesamt 45 Datensätze über drei Jahre ausgewertet werden konnten.
